# Consolidative chemotherapy after definitive concurrent chemoradiotherapy for esophageal squamous cell carcinoma patients: a population based cohort study

**DOI:** 10.1186/s12876-022-02464-x

**Published:** 2022-08-10

**Authors:** Chen-Yuan Lin, Ming-Yu Lien, Chi-Ching Chen, Hsin-Yuan Fang, Yu-Sen Lin, Chien-Kuang Chen, Jian-Xun Chen, Ting-Yu Lu, Tzu-Min Huang, Te-Chun Hsieh, Shung-Shung Sun, Chia-Chin Li, Chun-Ru Chien

**Affiliations:** 1 Division of Hematology and Oncology, Department of Internal Medicine, China Medical University Hospital, Taichung, Taiwan; 2 Department of Chest Surgery, China Medical University Hospital, Taichung, Taiwan; 3 Department of Nuclear Medicine and PET Center, China Medical University Hospital, Taichung, Taiwan; 4 Department of Biomedical Imaging and Radiological Science, China Medical University, Taichung, Taiwan; 5 Department of Radiation Oncology, China Medical University Hospital, Taichung, Taiwan; 6 School of Pharmacy, China Medical University, Taichung, Taiwan; 7 School of Medicine, College of Medicine, China Medical University, North District, No. 91 Hsueh-Shih Road, Taichung, 40402 Taiwan

**Keywords:** Consolidative chemotherapy, Definitive concurrent chemoradiotherapy, Esophageal squamous cell carcinoma

## Abstract

**Background:**

The role of consolidative chemotherapy (CCT) for locally advanced esophageal squamous cell carcinoma (LA-ESCC) patients treated with definitive concurrent chemoradiotherapy (dCCRT) is unclear. We aimed to compare the overall survival (OS) of those treated with vs without CCT via a population based approach.

**Methods:**

Eligible LA-ESCC patients diagnosed between 2011 and 2017 were identified via the Taiwan Cancer Registry. We used propensity score (PS) weighting to balance observable potential confounders between groups. The hazard ratio (HR) of death and incidence of esophageal cancer mortality (IECM) were compared between those with vs without CCT. We also evaluated the OS in supplementary analyses via alternative approaches.

**Results:**

Our primary analysis consisted of 368 patients in whom covariates were well balanced after PS weighting. The HR of death when CCT was compared to without was 0.67 (95% confidence interval 0.52–0.86, *P* = 0.002). The HR of IECM was 0.66 (*P* = 0.04). The HR of OS remained similarly in favor of CCT in supplementary analyses.

**Conclusions:**

We found that CCT was associated with significantly improved OS for LA-ESCC patients treated with dCCRT. Randomized controlled trials were needed to confirm this finding.

## Background

Esophageal cancer was one of the major causes of cancer mortality around the world including Taiwan [1, 2]. Squamous cell carcinoma (SqCC) was the common histology in the East whereas adenocarcinoma was more prevalent in the West [1, 2]. Most esophageal cancer patients were presented with locally advanced stage disease for whom definitive concurrent chemoradiotherapy (dCCRT) was commonly employed [3–6]. However, the long term survival outcomes of locally advanced esophageal cancer patients treated with dCCRT was still not satisfactory [7–10].

Treatment intensification via the use of consolidative (or called adjuvant) chemo therapy (CCT) after dCCRT for these patients may theoretically improve the outcome. However, it was not universally adopted as reflected in its mandatory use in some landmark randomized controlled trials (RCT) [8, 9] but excluded in the other RCTs [7, 10]. The role of CCT was also not clearly addressed in the current treatment guidelines [3–6]. A systematic review published in 2021 reported overall survival (OS) was significantly improved in the short term (1 year hazard ratio (HR) 0.542, *P* < 0.001) but not in the long term (5 year HR 0.923 *P* = 0.555) when CCT was compared to without CCT [11]. However, all the six studies regarding CCT in this systematic review were retrospective reviews from limited institutes [12–17]. Due to the lack of population based study, we aimed to compare the OS of locally advanced esophageal squamous cell carcinoma (LA-ESCC) patients treated with dCCRT with/without CCT via a population based approach.

## Material and methods

### Data source

Our study was a retrospective cohort study based on cancer registry. The analyzed data with personal identifiers removed was obtained from Health and Welfare Data Science Center (HWDC) database. The database included the Taiwan cancer registry (TCR), death registration, and reimbursement data for the whole Taiwan population provided by the Bureau of National Health Insurance (NHI). The TCR with comprehensive information (such as patient demographics, patient/disease/treatment characteristics) had been reported to be a good quality cancer registry [18]. This study had been approved by the Central Regional Research Ethics Committee at China Medical University Taichung Taiwan (CRREC-108-080 (CR2)).

### Study design, study population, and intervention

**Fig. 1:**
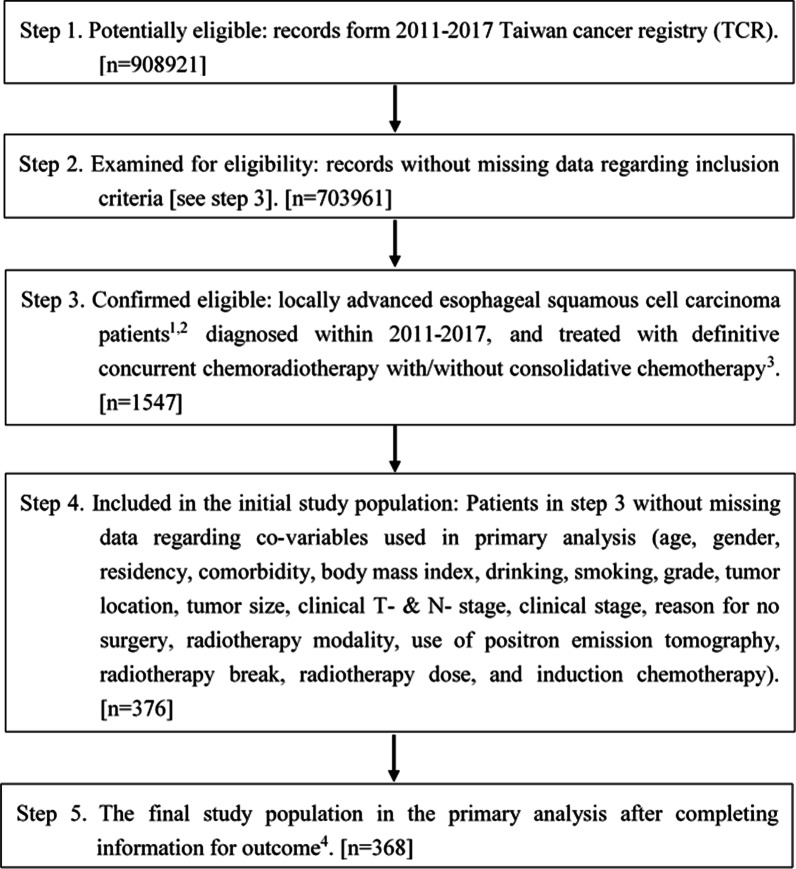
STROBE study flowchart and the number of individuals at each stage of the study. ^1^We only included those treated (class 1–2) to ensure data consistency. ^2^Clinical stage cT2-4N0M0 or cT1-4N+M0 for the 7th American Joint Committee on Cancer staging. ^3^50–70 Gy in 1.8–2 Gy/fraction. ^4^Without missing information in the TCR and death registry regarding survival status, and cause of death

The inclusion criteria of our study populations were (1) LA-ESCC adult (≥ 18 years old) patients diagnosed within 2011–2017 with locally-advanced stage defined as clinical stage cT2-4N0M0 or cT1-4N+M0 for the 7th American Joint Committee on Cancer staging; (2) treated with dCCRT without surgery according to the recording in TCR, with external beam radiotherapy 50–70 Gy in conventional fractionation. We excluded patients with multiple treatment records or prior other cancer(s) to ensure data quality. The study flowchart in concordant with STROBE statement [19] was depicted in Fig. 1.

The intervention (i.e., explanatory variable, with vs without CCT), the primary outcome (overall survival, OS) and the supplementary outcome (incidence of esophageal cancer mortality, IECM) were determined via the recordings of TCR or death registry. We defined the diagnostic date in TCR as the index date, and calculated OS/IECM from the index date to the death date (or Dec 31, 2019, i.e. the censoring date in death registry).

### Covariates

We collected covariates according to our clinical knowledge [20] via modification from recent relevant studies [21] and our clinical research experiences [22–24]. We used these covariates to adjust for potential nonrandomized treatment selection as defined as follows.

Patient demographics (age, gender, residency): age was classified as ≤ 58 or > 58 years old according a relevant study [21]. Patient residency region was classified as non-north or northern in Taiwan based on the variation in disease and care pattern we observed from clinical care and research experiences [24]. Patient characteristics (comorbidity, body mass index (BMI), drinking, smoking): comorbidity was determined by the modified Charlson comorbidity index score [25] and classified as with or without. BMI was classified as ≤ 18.5 or > 18.5 kg/m^2^ according to a relevant recent study [21]. The drinking and smoking were classified as no or yes.

Disease characteristics (grade, tumor location, tumor size, clinical T- & N-stage, clinical stage): Grade was classified as poorly or well/moderately differentiated. Tumor location was classified as upper, middle or lower. Tumor size was classified by a diameter ≤ 5 or > 5 cm. The clinical T-stage was classified as T1–T2 or T3–T4. The clinical N-stage was classified as N0 or N1–N2. The clinical stage was classified as II or III.

Diagnostic and treatment characteristics (use of positron emission tomography (PET), reason for no surgery, radiotherapy (RT) modality, RT break, RT dose, induction chemotherapy): The reason for “no surgery” was classified as either with contraindication or without contraindication (but patient refused or surgery was not planned). RT modality were classified as three-dimensional radiotherapy (3DCRT) or intensity-modulated radiotherapy (IMRT). The use of PET was classified as no or yes. For RT break, patients with radiotherapy prolongation was classified as ≤ 1 or > 1 week. RT dose was classified as low (50–50.4 Gy) or high (50.4–70 Gy) dose. The induction chemotherapy (ICT) was classified as with ICT (according to the recording in TCR plus systemic therapy at least 3 weeks before radiotherapy [11, 21]) or without ICT (patients started systemic therapy no earlier than 1 week before radiotherapy was started [11, 21]).

### Statistical analyses

In the primary analysis (PA), we adopted propensity score (PS) weighting (PSW) approach using overlap weight as the framework for analysis [26, 27]. To balance the measured potential confounders [28–30], we evaluated the probability of receiving CCT (vs. without CCT) as PS via a logistic regression model based on the above covariates, and then assessed the balance in covariates between groups via standardized difference [20, 30, 31]. In the weighted sample, we compared the hazard ratio (HR) of death between groups via Cox proportional hazards model for point estimation, and used the bootstrap method to estimate the 95% confidence interval (95% CI) [32–34]. We evaluated the impact of potential unmeasured confounder(s) via E-value as suggested in the literature [35]. We also estimated IECM via the competing risk approach [36] between groups in the weighted sample.

In the first supplementary analysis (SA-1), we used alternative analytic framework (PS matching, PSM) among the study population of primary analysis, and then constructed 1:1 PS matched cohorts to compare the HR of death between groups via a robust variance estimator [32]. In the second supplementary analysis (SA-2), we limited our study population to those with clinical response recorded in TCR and performed the PSW analysis in this subgroup to compare the HR of death as well as the response rate between groups.

All statistical analyses in this study were performed with the software SAS 9.4 (SAS Institute, Cary, NC) and R version 4.1.0 (R Development Core Team, R Foundation for Statistical Computing, Vienna, Austria).

## Results

### Study population in the primary analysis

Our study population consisted of 368 eligible locally advanced esophageal squamous cell carcinoma patients treated with dCCRT plus CCT (n = 103) or no CCT (n = 265) within 2011–2017 (Fig. 1). The patient characteristics were described in Table 1. Two covariates (tumor location, use of PET) were imbalanced before PS weighting, but all covariates achieved balance [20, 31] after PS weighting via overlap weights.

**Table 1:** Patient characteristics of the study population in the primary analysis

	Patient characteristics before PSW			Patient characteristics (%) after PSW^a^		
	CCT (n = 103)	Without CCT (n = 265)	Standardized difference^b^	CCT	Without CCT	Standardized difference^b^
	Number (%)^b^ or mean (SD)^b^	Number (%)^b^ or mean (SD)^b^				
Age (years)						
≤ 58	53 (51)	139 (52)	0.020	50	50	≈ 0
> 58	50 (49)	126 (48)		50	50	
Gender						
Female	5 (5)	12 (5)	0.015	5	5	≈ 0
Male	98 (95)	253 (95)		95	95	
Residency						
Non-north	77 (75)	185 (70)	0.111	73	73	≈ 0
North	26 (25)	80 (30)		27	27	
Comorbidity						
Without	91 (88)	233 (88)	0.013	89	89	≈ 0
With^c^	12 (12)	32 (12)		11	11	
BMI (kg/m^2^)						
≤ 18.5	22 (21)	62 (23)	0.049	21	21	≈ 0
> 18.5	81 (79)	203 (77)		79	79	
Drinking						
No	14 (14)	46 (17)	0.104	14	14	≈ 0
Yes	89 (86)	219 (83)		86	86	
Smoking						
No	10 (10)	43 (16)	0.195	11	11	≈ 0
Yes	93 (90)	222 (84)		89	89	
Grade						
Poorly	34 (33)	59 (22)	0.242	30	30	≈ 0
Well/moderately differentiated	69 (67)	206 (78)		70	70	
Tumor location						
Upper	56 (54)	97 (37)		50	50	
Middle	34 (33)	122 (46)	0.269	36	36	≈ 0
Lower	13 (13)	46 (17)	0.133	14	14	≈ 0
Tumor size (cm)						
≤ 5 cm	43 (42)	99 (37)	0.090	41	41	≈ 0
> 5 cm	60 (58)	166 (63)		59	59	
Clinical T-stage						
T1–T2	10 (10)	32 (12)	0.076	10	10	≈ 0
T3–T4	93 (90)	233 (88)		90	90	
Clinical N-stage						
N0	9 (9)	23 (9)	0.002	9	9	≈ 0
N1-N2	94 (91)	242 (91)		91	91	
Clinical stage						
II	11 (11)	30 (11)	0.020	12	12	≈ 0
III	92 (89)	235 (89)		88	88	
Reason for no surgery						
Without contraindication	99 (96)	248 (94)	0.115	95	95	≈ 0
With contraindication	4 (4)	17 (6)		5	5	
RT modality						
3DCRT	7 (7)	7 (3)	0.197	5	5	≈ 0
IMRT	96 (93)	258 (97)		95	95	
Use of PET						
No	45 (44)	77 (29)	0.308	37	37	≈ 0
Yes	58 (56)	188 (71)		63	63	
RT break						
≤ 1 week	79 (77)	197 (74)	0.055	76	76	≈ 0
> 1 week	24 (23)	68 (26)		24	24	
RT dose						
Low	25 (24)	88 (33)	0.198	26	26	≈ 0
High	78 (76)	177 (67)		74	74	
Induction chemotherapy						
Without	98 (95)	258 (97)	0.117	96	96	≈ 0
With	5 (5)	7 (3)		4	4	

3DCRT, three-dimensional radiotherapy; BMI, Body Mass Index; CCT, consolidative chemotherapy; IGRT, image-guided radiotherapy; IMRT, intensity-modulated radiotherapy; PET, positron emission tomography; PSW, Propensity Score (PS) Weighting; RT, radiotherapy; SD, standard deviation

^a^Weighted proportion for each group

^b^Rounded

^c^Modified Carlson comorbidity score ≥ 1

### Primary analysis

**Fig. 2:**
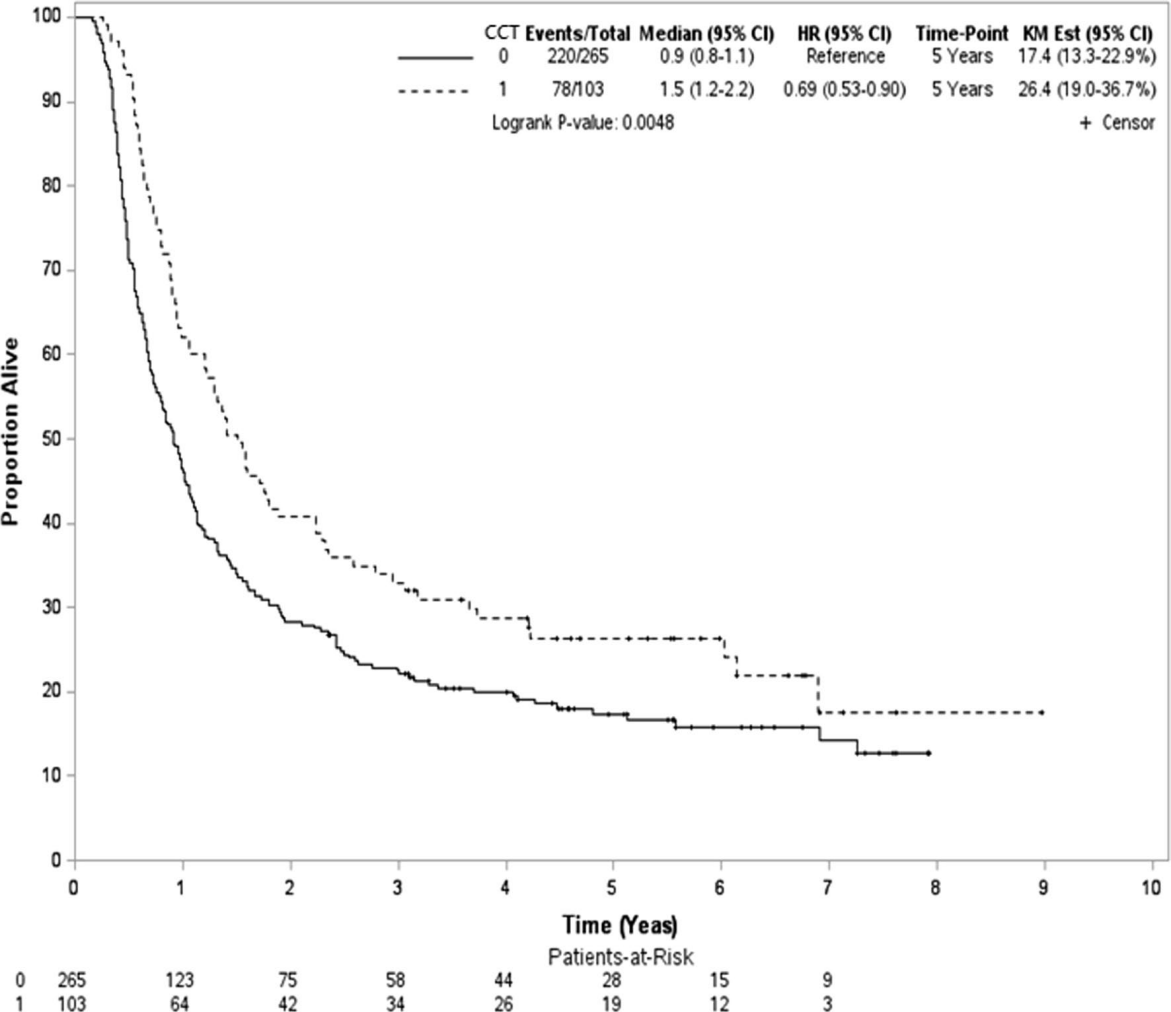
Kaplan–Meier unadjusted overall survival curve (in years) in the primary analysis. CCT, consolidative chemotherapy

**Fig. 3:**
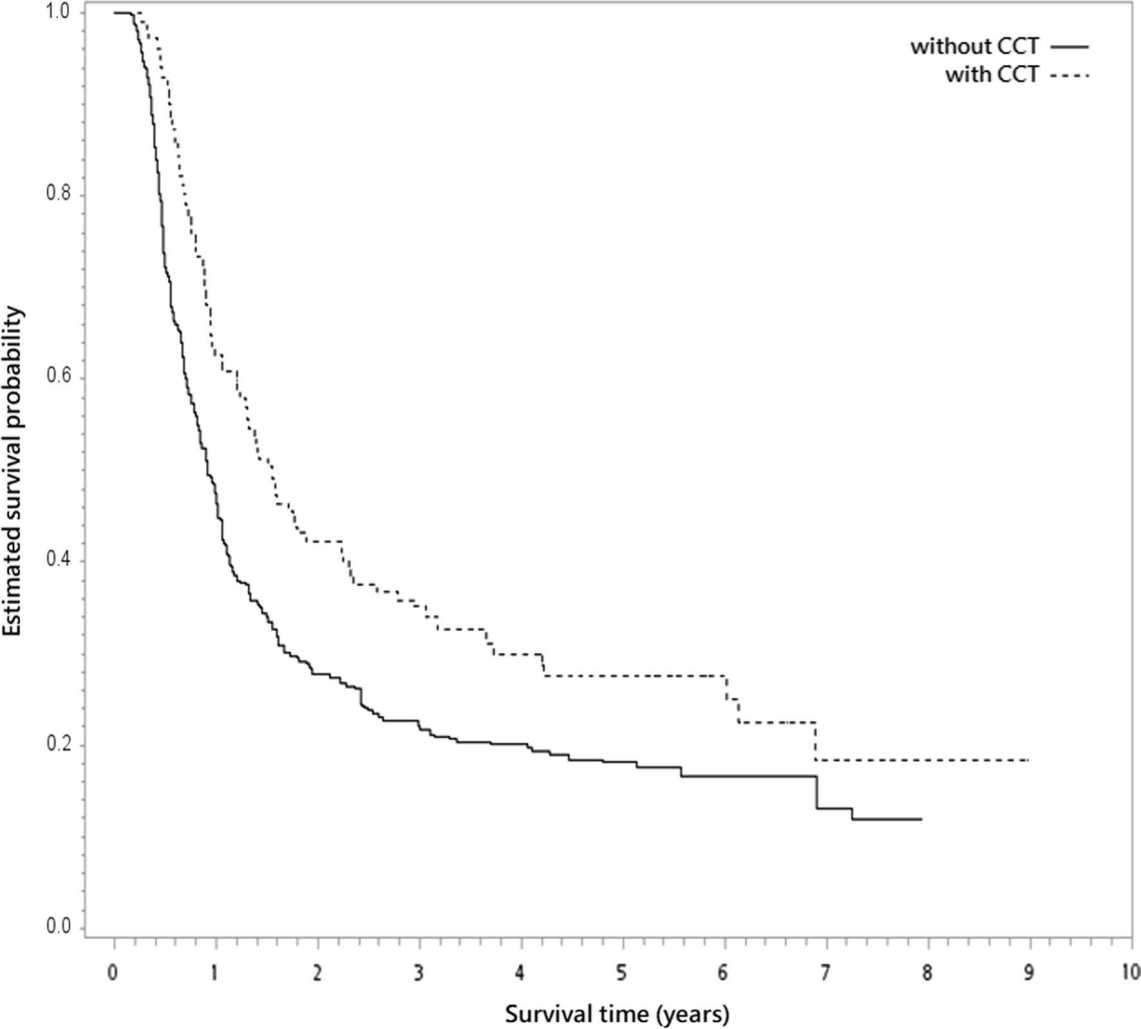
The overlap weights adjusted overall survival curve (in years) in the primary analysis. CCT, consolidative chemotherapy

During the follow-up period with median follow-up 12 months (range 2–107 months), 298 deaths were observed (78 and 220 for patients with CCT or without CCT respectively). For survivors, the median follow-up was 63 months (range 28–107). In the unadjusted analysis, the 5-year OS rate was 26% and 17% for those with CCT and without CCT respectively (log-rank test, *P* = 0.005; Fig. 2). In the PSW analysis, the 5-year PSW-adjusted OS rate between groups were 28% (with CCT) and 18% (without CCT) respectively. The overlap weights adjusted OS curve was shown in Fig. 3. When CCT was compared to without CCT, the PSW adjusted HR of death was 0.67 (95% confidence interval (95% CI) 0.52–0.86, *P* = 0.002). The observed HR 0.67 for OS could be explained by an unmeasured confounder associated with both selection of treatment and survival by a risk ratio of 1.97 (E-value) fold each, but weaker confounding factors could not. The result was also in favor of CCT for IECM (HR = 0.66, 95% CI 0.44–0.99, *P* = 0.04).

### Supplementary analyses (SA-1, SA-2)

**Fig. 4:**
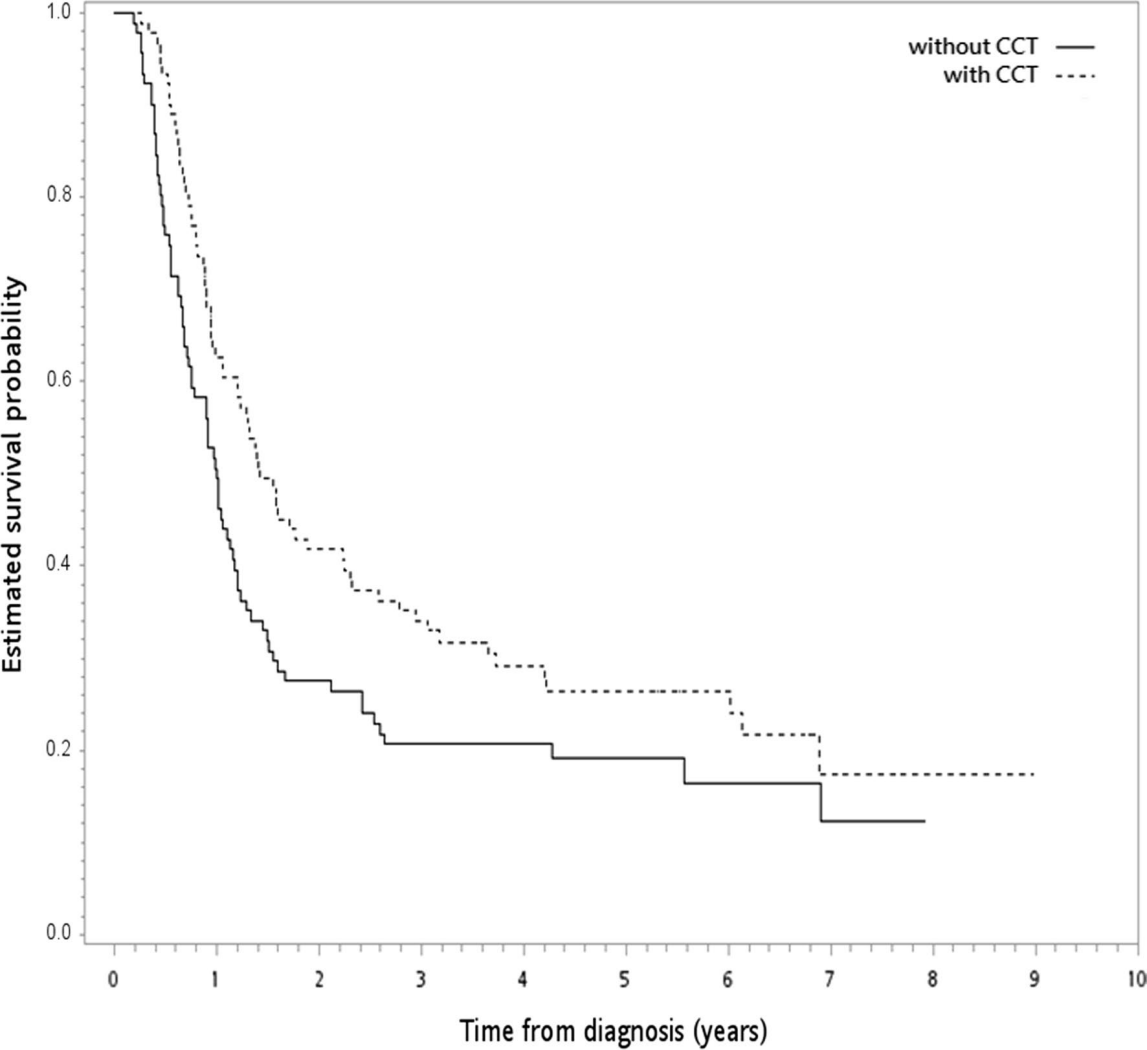
Kaplan–Meier survival curve (in years) for the PS-matched subgroup (SA-1). CCT, consolidative chemotherapy

In the SA-1, we achieved all covariates balance (standardized difference ≤ 0.25 [31]) after PSM in the PS-matched subgroup (n = 182; Table 2). The 5-year OS rate was 26% (with CCT) and 19% (without CCT) respectively. The Kaplan Meier OS curve was shown in Fig. 4. There was also statistically significant difference for OS (HR = 0.69, 95% CI 0.50–0.94, *P* = 0.02).

**Table 2:** SA-1: patient characteristics of the PS-matched subgroup

	CCT (n = 91)		Without CCT (n = 91)		Standardized difference^a^
	Number or mean (SD)^a^	(%)^a^	Number or mean (SD)^a^	(%)^a^	
Age (years)					
≤ 58	47	(52)	44	(48)	0.066
> 58	44	(48)	47	(52)	
Gender					
Female	5	(5)	3	(3)	0.107
Male	86	(95)	88	(97)	
Residency					
Non-north	66	(73)	70	(77)	0.101
North	25	(27)	21	(23)	
Comorbidity					
Without	79	(87)	83	(91)	0.141
With^b^	12	(13)	8	(9)	
BMI (kg/m^2^)					
≤ 18.5	20	(22)	14	(15)	0.170
> 18.5	71	(78)	77	(85)	
Drinking					
No	13	(14)	15	(16)	0.061
Yes	78	(86)	76	(84)	
Smoking					
No	10	(11)	10	(11)	0
Yes	81	(89)	81	(89)	
Grade					
Poorly	28	(31)	23	(25)	0.123
Well/moderately differentiated	63	(69)	68	(75)	
Tumor location					
Upper	46	(51)	44	(48)	
Middle	32	(35)	35	(39)	0.068
Lower	13	(14)	12	(13)	0.032
Tumor size (cm)					
≤ 5 cm	38	(42)	43	(47)	0.111
> 5 cm	53	(58)	48	(53)	
Clinical T-stage					
T1–T2	10	(11)	9	(10)	0.036
T3–T4	81	(89)	82	(90)	
Clinical N-stage					
N0	9	(10)	8	(9)	0.038
N1–N2	82	(90)	83	(91)	
Clinical stage					
II	11	(12)	10	(11)	0.034
III	80	(88)	81	(89)	
Reason for no surgery					
Without contraindication	87	(96)	87	(96)	0
With contraindication	4	(4)	4	(4)	
RT modality					
3DCRT	6	(7)	3	(3)	0.152
IMRT	85	(93)	88	(97)	
Use of PET					
No	34	(37)	36	(40)	0.045
Yes	57	(63)	55	(60)	
RT break					
≤ 1 week	69	(76)	70	(77)	0.026
> 1 week	22	(24)	21	(23)	
RT dose					
Low	23	(25)	22	(24)	0.025
High	68	(75)	69	(76)	
Induction chemotherapy					
Without	87	(96)	86	(95)	0.051
With	4	(4)	5	(5)	

3DCRT, three-dimensional radiotherapy; BMI, Body Mass Index; CCT, consolidative chemotherapy; IGRT, image-guided radiotherapy; IMRT, intensity-modulated radiotherapy; PET, positron emission tomography; RT, radiotherapy; SD, standard deviation

^a^Rounded

^b^Modified Carlson comorbidity score ≥ 1

In the SA-2, covariate balance was also achieved after PSW although some were imbalanced before PSW as shown in Table 3 (n = 246). Comparisons between groups revealed significantly better OS for those with CCT versus without CCT [PSW adjusted HR 0.68 (95% CI 0.49–0.92, *P* = 0.013). The crude response rate (77% vs. 68%) was higher for those with vs without CCT, but without statistical significance (PSW adjusted odds ratio (OR) 1.61, 95% CI 0.62–2.60, *P* = 0.23).

**Table 3:** SA-2: patient characteristics of the subgroup with clinical response recorded

	Patient characteristics before PSW			Patient characteristics (%) after PSW^a^		
	CCT (n = 79)	Without CCT (n = 167)	Standardized difference^b^	CCT	Without CCT	Standardized difference^b^
	Number (%)^b^ or mean (SD)^b^	Number (%)^b^ or mean (SD)^b^				
Age (years)						
≤ 58	42 (53)	90 (54)	0.015	51	51	≈ 0
> 58	37 (47)	77 (46)		49	49	
Gender						
Female	4 (5)	7 (4)	0.042	5	5	≈ 0
Male	75 (95)	160 (96)		95	95	
Residency						
Non-north	60 (76)	119 (71)	0.107	74	74	≈ 0
North	19 (24)	48 (29)		26	26	
Comorbidity						
Without	69 (87)	146 (87)	0.003	88	88	≈ 0
With^c^	10 (13)	21 (13)		12	12	
BMI (kg/m^2^)						
≤ 18.5	15 (19)	36 (22)	0.064	19	19	≈ 0
> 18.5	64 (81)	131 (78)		81	81	
Drinking						
No	11 (14)	22 (13)	0.022	14	14	≈ 0
Yes	68 (86)	145 (87)		86	86	
Smoking						
No	8 (10)	20 (12)	0.059	11	11	≈ 0
Yes	71 (90)	147 (88)		89	89	
Grade						
Poorly	25 (32)	40 (24)	0.172	31	31	≈ 0
Well/moderately differentiated	54 (68)	127 (76)		69	69	
Tumor location						
Upper	43 (54)	65 (39)		50	50	
Middle	25 (32)	75 (45)	0.275	35	35	≈ 0
Lower	11 (14)	27 (16)	0.063	15	15	≈ 0
Tumor size (cm)						
≤ 5 cm	34 (43)	61 (37)	0.133	43	43	≈ 0
> 5 cm	45 (57)	106 (63)		57	57	
Clinical T-stage						
T1–T2	7 (9)	17 (10)	0.045	8	8	≈ 0
T3–T4	72 (91)	150 (90)		92	92	
Clinical N-stage						
N0	7 (9)	8 (5)	0.162	7	7	≈ 0
N1–N2	72 (91)	159 (95)		93	93	
Clinical stage						
II	9 (11)	14 (8)	0.101	10	10	≈ 0
III	70 (89)	153 (92)		90	90	
Reason for no surgery						
Without contraindication	75 (95)	154 (92)	0.111	94	94	≈ 0
With contraindication	4 (5)	13 (8)		6	6	
RT modality						
3DCRT	7 (9)	4 (2)	0.283	5	5	≈ 0
IMRT	72 (91)	163 (98)		95	95	
Use of PET						
No	34 (43)	42 (25)	0.384	35	35	≈ 0
Yes	45 (57)	125 (75)		65	65	
RT break						
≤ 1 week	63 (80)	125 (75)	0.117	79	79	≈ 0
> 1 week	16 (20)	42 (25)		21	21	
RT dose						
Low	19 (24)	54 (32)	0.185	26	26	≈ 0
High	60 (76)	113 (68)		74	74	
Induction chemotherapy						
Without	74 (94)	162 (97)	0.159	95	95	≈ 0
With	5 (6)	5 (3)		5	5	

3DCRT, three-dimensional radiotherapy; BMI, Body Mass Index; CCT, consolidative chemotherapy; IGRT, image-guided radiotherapy; IMRT, intensity-modulated radiotherapy; PET, positron emission tomography; PSW, propensity score weighting; RT, radiotherapy; SD, standard deviation

^a^Weighted proportion for each group

^b^Rounded

^c^Modified Carlson comorbidity score ≥ 1

## Discussion

In our population based cohort study, we found that CCT was associated with significantly improved OS for LA-ESCC patients treated with dCCRT. This was the 1st population based study to our knowledge.

In our mind, our results were compatible with the results in the above-mentioned systematic review in that the point estimate of HR for OS was in favor of CCT [11]. In another systematic review published in 2021 (not limited to SqCC but consisted of mainly SqCC patients) [37], favorable OS (HR 0.72; 95% CI 0.59–0.86, *P* < 0.001) and response rate (OR 1.44; 95% CI 0.62–3.35, *P* = 0.393) were reported. Our results were relatively close to these results. When we looked at the relevant individual studies [12–17] included in the above systematic review [11], the details were summarized below. Wu et al. compared 67 patients in the CCT group vs 142 patients in control group treated at a single institute and found CCT improved the overall survival with HR 0.67 [12]. In 524 PS matched patients treated from two institutes, Liu et al. reported OS HR 0.92 [13]. Chen et al. investigated 187 patients (89 with CCT whereas 98 without CCT) treated at two institutes and reported OS HR 0.971 in the univariate analyses [14]. Among 124 patients (65 with CCT and 59 without CCT) treated with dCCRT from a single institute, Chen et al. reported the median OS to be 19 months (without CCT) vs. 25 months (with CCT) [15]. From 73 patients treated with dCCRT at three institutes, Koh et al. reported CCT improved OS (3-year, 24.2% vs. 11.8%, *P* = 0.004) [16]. Among 222 patients (113 with CCT and 109 without CCT) treated with dCCRT from a single institute, Zhang et al. reported the median OS to be 18 months (without CCT) vs. 33 months (with CCT) (*P* = 0.003) [17]. Therefore, our results were compatible with most of these studies [12, 15–17] in favor of CCT. Furthermore, our study utilized papulation-based cancer registry so were more representative than these studies relied on patients from one ~ three institutes.

The interpretation of our results seems strait forward because the outcomes were improved after treatment intensification. However, RCT were needed to confirm our finding because negative results of CCT had been reported in other disease sites such as lung cancer [38]. The generalizability of our finding to current practice was also not clear in the era of immunotherapy [39, 40].

There were several limitations in our study. First of all, there were always concerns regarding potential unmeasured confounder(s) in non-randomized studies although we had used propensity score to adjust for measured covariates and used *E* value to address the impact of the potential unmeasured confounders. For example, radiotherapy volume or chemotherapy regimens or cycles may be imbalance between groups but were not considered in our study due to data limitation. Therefore, we reported the *E *value (1.97) as suggested in the literature to evaluate the potential impact of possible unmeasured confounder(s) [35]. Secondly, other endpoints such as progression free survival or quality of life may also be important but were not investigated due to data limitation as well.

## Conclusions

We found that CCT was associated with significantly improved OS for LA-ESCC patients treated with dCCRT. RCT was needed to confirm this finding.

## Data Availability

The data that support the findings of this study are available from [Health and Welfare Data Science Center, Ministry of Health and Welfare, Executive Yuan, Taiwan] but restrictions apply to the availability of these data, which were used under license for the current study, and so are not publicly available. Data are however available from the authors upon reasonable request and with permission of [Health and Welfare Data Science Center, Ministry of Health and Welfare, Executive Yuan, Taiwan].

## Abbreviations

95% CI: 95% Confidence interval
3DCRT: Three-dimensional radiotherapy
BMI: Body Mass Index
CCT: Chemo therapy
dCCRT: Definitive concurrent chemoradiotherapy
HR: Hazard ratio
ICT: Induction chemotherapy
HWDC: Health and Welfare Data Science Center
IECM: Incidence of esophageal cancer mortality
IMRT: Intensity-modulated radiotherapy
LA-ESCC: Locally advanced esophageal squamous cell carcinoma
NHI: National Health Insurance
OS: Overall survival
PA: Primary analysis
PET: Positron emission tomography
PS: Propensity Score
PSW: Propensity Score Weighting
RCT: Randomized controlled trial
RT: Radiotherapy
SA-1: The first supplementary analysis
SA-2: The second supplementary analysis
SqCC: Squamous cell carcinoma
TCR: Taiwan cancer registry
